# Free Time and Physical Activity Among Americans 15 Years or Older: Cross-Sectional Analysis of the American Time Use Survey

**DOI:** 10.5888/pcd16.190017

**Published:** 2019-09-26

**Authors:** Roland Sturm, Deborah A. Cohen

**Affiliations:** 1 RAND Corporation, Santa Monica, California

## Abstract

**Introduction:**

Many Americans fail to meet physical activity guidelines. We investigated whether this failure is due in part to a lack of free time.

**Methods:**

We analyzed data from the American Time Use Survey, 2014 through 2016, with 32,048 respondents aged 15 years or older, categorizing every activity during a 24-hour period. Free or leisure time includes time spent socializing, being entertained, in sports and recreation activities, volunteering, in religious activities, taking classes for personal interest, and in associated travel time. Working in the labor market, education (unless only for personal interest), household work and home production (cooking, cleaning, child care, shopping), or self-care (sleeping, eating, grooming) are not free time. We stratified by sociodemographic characteristics, health, and body mass index, and we calculated descriptive statistics adjusted for the multistage sampling design.

**Results:**

Americans averaged more than 5 hours (>300 minutes) of free time per day; no subgroup reported having less than 4.5 hours (270 minutes) of free time. Men had more free time (mean [standard deviation], 356 [3] min/d) and spent more on leisure time physical activity (mean [SD], 24 [3] min/d) than women did (free time mean [SD], 318 [2] min/d, *P* < .001; and leisure time physical activity mean [SD], 14 [1] min/d, *P* < .001). Compared with those with a higher income and a college education, those with income below 185% of federal poverty guidelines and those with a high school education reported more free time but spent more time on television, movies, and other screen time and less on physical activity (all comparisons *P* < .001).

**Conclusion:**

Lack of free time is not responsible for low levels of leisure time physical activity at the population level.

**Summary**

**What is already known on this topic?**

Many Americans are insufficiently active, and there are disparities across sociodemographic groups, which may be due in part to a lack of free time.

**What is added by this report?**

On average, Americans in all sociodemographic groups have large amounts of free time, with no group averaging less than 4.5 hours per day. There is no direct relationship between free time and physical activity. Instead, some of the most active groups (eg, college educated, higher income) report less free time than other groups, but more physical activity and less screen time.

**What are the implications for public health practice?**

Increasing people’s awareness of how they actually use time and messages targeting screen time are promising.

## Introduction

Physical activity has multiple health benefits: lower risk and severity of chronic diseases (including heart disease, diabetes, and some cancers), lower mortality rates, and improved mental health and physical well-being (1). Yet many Americans do not achieve levels consistent with guideline recommendations. Estimates of adults meeting guidelines vary depending on assessment methods, but according to the latest National Health Interview Survey, about 53% meet the aerobic guidelines of 150 min/wk of leisure time moderate or vigorous activity (2). Insufficient levels of physical activity may account for 8% of annual deaths in the United States (1,3).

Physical activity requires motivation, but it also requires time. Many studies have documented low levels of physical activity but not how this activity fits (or does not fit) into a person’s day. The American Cancer Society’s guidelines on nutrition and physical activity echo common beliefs by claiming that “reduced leisure time . . . contribute[s] to reduced levels of physical activity” (4). Time constraints can limit physical activity and possibly even contribute to disparities across sociodemographic groups.

Neighborhood parks are settings designed to support leisure time physical activity and are free and open to all, yet repeated observations have documented that, on a national level, there are substantial disparities in their use by sex and by neighborhood socioeconomic status (5,6). Parks in low-income neighborhoods are used less than those in high-income neighborhoods (7), and females of all ages use them less than males (5). The degree to which these disparities reflect constraints on free time is not known.

Time use data provide a way to understand how physical activity fits into a person’s day. We revisited questions about free time and physical activity through the US Census Bureau’s American Time Use Survey (ATUS) for 2014 through 2016. These public use data are unlike most others: ATUS uses a time-diary approach where respondents recall their activities sequentially for a day, while most other surveys like the Behavioral Risk Factor Surveillance System ask respondents to estimate frequency and duration of physical activity during a typical week. ATUS classifies these time-constrained activity reports in categories like work, care for family and children, and self-care. Free time comprises entirely discretionary activities not essential for daily survival (8). We analyzed the ATUS data set to test whether free time was associated with physical activity.

## Methods

ATUS is a continuous survey that determines how Americans spend their time (9). ATUS samples nationally representative households and conducts interviews throughout the year. One individual aged 15 years or older is randomly chosen from each household and assigned a day of the week about which to report what he or she did for 24 hours.

ATUS assigned codes for each reported activity in a 24-hour day, which were categorized into 17 broad categories: 1) personal care; 2) household activities; 3) caring for household members; 4) caring for nonhousehold members; 5) work; 6) education; 7) consumer purchases; 8) professional and personal care services; 9) household services; 10) government services and civil obligations; 11) eating and drinking; 12) socializing, relaxing, and leisure; 13) sports (both active and spectator), exercise, and recreation; 14) religious activities; 15) volunteer activities; 16) telephone calls; and 17) traveling. We defined free time as socializing, relaxing, and leisure; sports; exercise and recreation; volunteer activities; religious activities; taking classes for personal interest (but not if part of degree program, certification, or licensure) (part of education); extracurricular activities for students (part of education); and travel associated with those leisure-time activities (part of traveling). We subdivided free time into 4 categories: 1) screen time, 2) physical activity time, 3) travel related to free time, and 4) “other” free time activities. Screen time included television watching, games, and computer use (unless for work or other categories); screen time captures electronic activities at home. Physical activity time includes active (not spectator) sports, exercise, and recreational activities. Because we only included physical activity during free time, this excludes utilitarian physical activity (ie, physical effort as part of work or household tasks). “Other” free time activities include socializing and communicating, attending or hosting social events, arts and entertainment other than sports, visiting museums, religious activities, and classes for personal interest. We coded whether physical activity was reported to be outdoors to distinguish it from exercising at a gym or home, but location information was incomplete for about 15% of physical activity time.

We analyzed data for 2014 through 2016, which included information on health status, body mass index, and income not collected in other years. Estimates were adjusted for 3 aspects of the sampling and data collection process: 1) some demographic groups were oversampled to ensure adequate sample size for stratified estimates; 2) differential sampling of days (25% of the sample were assigned to report on each of the 2 weekend days and 10% of the sample to each of the 5 weekdays); and 3) differential response rates of demographic groups and days of the week (10). Estimates were weighted to ensure national representativeness and that days of the week were equally represented in spite of differing response rates. We stratified income by being either below or at or above 185% of the federal poverty guidelines, a threshold used for determination of eligibility in several government support programs. The results reflect the average number of minutes in specific activities in a typical day for the specified population. *P* values were calculated for *t* tests (for 2 group comparisons [eg, men and women]) or *F* tests (for equality across multiple groups). Analyses were conducted with Stata version 15.0 (StataCorp LLC).

## Results

### Population characteristics

The ATUS for 2014 through 2016 had 32,048 respondents (Table 1). Missing values on income, health status, or body mass index reduced sample sizes when those variables were analyzed. The largest number of missing values was for body mass index (2,147 of 32,048 [6.7%]).

**Table 1:** Characteristics of Respondents (N = 32,048), American Time Use Survey, 2014–2016^a^

Characteristic	Men, No. (%)	Women, No. (%)	All, No. (%)
**Age, y**			
15–24	1,435 (10)	1,466 (8)	2,901 (9)
25–60	8,698 (61)	10,490 (59)	19,188 (60)
>60	4,015 (28)	5,944 (33)	9,959 (31)
Total	14,148 (100)	17,900 (100)	32,048 (100)
**Race/ethnicity**			
White, non-Hispanic	9,297 (66)	11,613 (65)	20,910 (65)
Hispanic	2,205 (16)	2,554 (14)	4,759 (15)
Black	1,828 (13)	2,716 (15)	4,544 (14)
Other	818 (6)	1,017 (6)	1,835 (6)
Total	14,148 (100)	17,900 (100)	32,048 (100)
**Education level (respondents aged >24 y)**			
Less than high school diploma	1,989 (14)	2,139 (12)	4,128 (13)
Completed high school	3,378 (24)	4,419 (25)	7,797 (24)
Some post–high school education	3,698 (26)	5,148 (29)	8,846 (28)
Completed college	5,083 (36)	6,194 (35)	11,277 (35)
Total	14,148 (100)	17,900 (100)	32,048 (100)
**Income, % of federal poverty guidelines**			
≥185	9,562 (69)	10,601 (61)	20,163 (65)
<185	4,300 (31)	6,749 (39)	11,049 (35)
Total	13,862 (100)	17,350 (100)	31,212 (100)
**Body mass index^b^**			
18.5–24.9	3,779 (28)	6,379 (39)	10,158 (34)
25.0–29.9	5,720 (42)	4,941 (31)	10,661 (36)
30.0–34.9	2,802 (20)	2,751 (17)	5,553 (19)
≥35.0	1,407 (10)	2,122 (13)	3,529 (12)
Total	13,708 (100)	16,193 (100)	29,901 (100)
**Self-reported health**			
Excellent	2,572 (18)	3,138 (18)	5,710 (18)
Very good	4,824 (34)	5,899 (33)	10,723 (34)
Good	4,486 (32)	5,583 (31)	10,069 (32)
Fair	1,595 (11)	2,365 (13)	3,960 (12)
Poor	526 (4)	767 (4)	1,293 (4)
Total	14,003 (100)	17,752 (100)	31,755 (100)

a Differences in totals in different categories are due to missing responses.

b Body mass index calculated as weight in kilograms divided by the square of height in meters.

### Free time minutes and demographic characteristics

Men reported more free time than women did (mean [standard deviation (SD)], 356 [3] min/d vs 318 [2] min/d, *P* < .001) and spent 10 min/d more on physical activity (mean [SD], 24 [3] min/d vs 14 [1] min/d, *P* < .001) (Table 2). However, men spent essentially all of the additional free time (36 of the 38 minutes) on screen time (mean [SD], 211 [2] min/d for men vs 175 [2] min/d for women; *P* < .001) and less time across the range of “other” free time activities compared with women. Men reported about 11% more free time than women did, but men reported about 20% more screen time than women did.

**Table 2:** Average Daily Minutes of Free Time, Screen Time, Leisure Time Physical Activity, and Physical Activity Outdoors Among Americans Aged 15 Years or Older (N = 32,048), American Time Use Survey, 2014–2016^a^

Characteristic	Free Time, Min/d			Screen Time, Min/d			Leisure Time Physical Activity, Min/d			Physical Activity Outdoors,^b^ Min/d		
	Men	Women	*P* Value	Men	Women	*P* Value	Men	Women	*P* Value	Men	Women	*P* Value
**Total**	356	318	<.001	211	175	<.001	24	14	<.001	13	6	<.001
**Age, y**												
15–24	377	300	<.001	210	161	<.001	39	21	<.001	18	8	<.001
25–60	304	271	<.001	179	145	<.001	20	13	<.001	11	6	<.001
>60	466	426	<.001	288	247	<.001	21	11	<.001	13	5	<.001
*P* value	<.001	<.001		<.001	<.001		<.001	<.001		.004	.002	
**Race/ethnicity^c^**												
White non-Hispanic	356	326	<.001	210	179	<.001	24	15	<.001	14	6	<.001
Hispanic	320	270	<.001	186	146	<.001	22	12	<.001	11	6	<.001
Black	423	355	<.001	261	212	<.001	22	7	<.001	9	2	<.001
Other	324	276	<.001	193	146	<.001	26	16	.001	12	6	<.001
*P* value	<.001	<.001		<.001	<.001		.35	<.001		.009	<.001	
**Education level (respondents aged >24 y)^d^**												
Less than high school diploma	398	358	<.001	254	218	<.001	15	8	<.001	10	5	<.001
Completed high school	380	355	<.001	247	218	<.001	17	8	<.001	12	4	<.001
Some post–high school education	345	317	<.001	215	179	<.001	20	11	<.001	12	5	<.001
Completed college	315	286	<.001	164	133	<.001	25	18	<.001	13	7	<.001
*P* value	<.001	<.001		<.001	<.001		.03	<.001		.33	<.001	
**Income, % of federal poverty guidelines^c^**												
≥185	339	303	<.001	194	159	<.001	26	17	<.001	14	7	<.001
<185	386	336	<.001	244	200	<.001	17	9	<.001	9	4	<.001
*P* value	<.001	<.001		<.001	<.001		<.001	<.001		<.001	<.001	
**Body mass index^e^**												
18.5–24.9	355	300	<.001	200	156	<.001	27	18	<.001	14	8	<.001
25.0–29.9	347	323	<.001	202	180	<.001	26	14	<.001	15	6	<.001
30.0–34.9	361	341	.01	223	195	<.001	19	9	<.001	10	4	<.001
≥35	375	342	.002	244	207	<.001	15	6	<.001	10	3	<.001
*P* value	<.001	<.001		<.001	<.001		.02	<.001		<.001	<.001	
**Self-reported health**												
Excellent	317	296	<.001	162	140	<.001	35	25	<.001	17	10	<.001
Very good	336	306	.004	190	160	<.001	27	15	<.001	15	7	<.001
Good	358	311	<.001	218	178	<.001	18	10	<.001	11	4	<.001
Fair	421	368	<.001	287	228	<.001	14	6	<.001	8	3	<.001
Poor	523	406	<.001	367	278	<.001	9	4	.02	6	2	.02
*P* value	<.001	<.001		<.001	<.001		<.001	<.001		<.001	<.001	
**Weekday/weekend**												
Weekday	316	288	<.001	191	166	<.001	22	13	<.001	11	5	<.001
Saturday or Sunday	454	391	<.001	261	198	<.001	28	16	<.001	17	8	<.001
*P* value	<.001	<.001		<.001	<.001		<.001	.003		<.001	<.001	

a Weighted means based on 32,048 respondents (14,148 male, 17,900 female) to American Time Use Survey, 2014–2016.

b Only includes activities explicitly coded as “outdoors away from home.” Many activities that are likely to be outdoors were coded as “other location.”

c Discrepancies in data between this table and the figures in this article are due to rounding.

d Respondents younger than 25 years (1,435 male, 1,466 female) were excluded in the stratification by education (only for that variable) because their educational achievement may not be complete.

e Body mass index calculated as weight in kilograms divided by the square of height in meters.

The oldest group reported the highest levels of free time, and the youngest group reported the highest level of physical activity (Table 2). By race/ethnicity, there were large differences. Black men had about an hour more daily free time than white non-Hispanic white men (mean [SD], 423 [8] min/d vs 356 [3] min/d, *P* < .001) and 1.5 hours more free time than Hispanic men (mean [SD], 320 [6] min/d, *P* < .001) or other race groups (mean [SD], 324 [11] min/d, *P* < .001). This was paralleled in screen time. However, there were no differences in leisure time physical activity levels by race/ethnicity among men (*P* = .35); almost all other comparisons were significant at a *P* value of less than .001 (Table 2). For both free time and screen time, the same pattern across race/ethnicity held for women (highest for black women, lowest for Hispanic and other); overall, women had less free time and less screen time than men did. However, black women engaged only in about half the physical activity time compared with women in other racial/ethnic groups (*P* < .001).

### Free time, education, and poverty

We excluded the youngest category (<25 years) in the stratification by education achievement because their education may not yet have been completed. For respondents 25 or older, the relationship between education and free and screen time was monotonic; less education was associated with more free and screen time among both men and women (*P* < .001). The relationship was inverted for leisure time physical activity, with higher education associated with more time spent on physical activity (*P* = .03 for men, *P* < .001 for women).

Stratifying by income (either below or at or above 185% of federal poverty guidelines) resulted in the same pattern: free and screen time were higher among lower-income groups, yet time engaged in physical activity level was significantly lower (*P* < .001 for all comparisons).

### Free time, body mass index, health, and day of the week

Regarding the relationship between time use and body mass index or self-reported health, heavier or less healthy respondents reported more free time and more screen time but less engagement in physical activity. Free time, screen time, and physical activity time were higher on weekends than during the week.

### Free time, sex, income, and race/ethnicity

**Figure 1:**
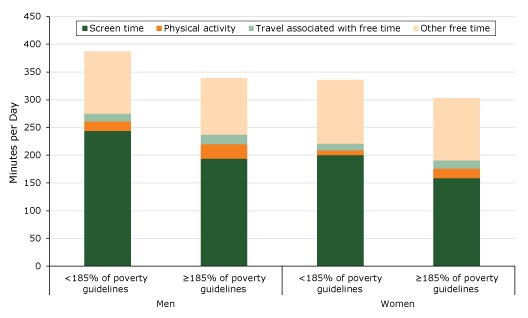
Distribution of free time, in minutes per day, by income (<185% or ≥185% of federal poverty guidelines) and sex (N = 32,048), American Time Use Survey, 2014–2016. Test of equality: *P* < .001 for all of tests of equality of either the proportion of screen time or of physical activity: lower income men vs higher income men, lower income women vs higher income women, lower income men vs lower income women; higher income men vs higher income women. Discrepancies in data between this figure and Table 2 of this article are due to rounding.

Across income groups, men spent a mean (SD) of 6.6% (0.2) of their free time on physical activity, and women spent a mean (SD) of 5.0% (0.11). Higher-income men and women spent a larger share of their free time on physical activity and less on screen time than did lower-income women (*P* < .001 for all tests) (Figure 1).

**Table:** 

Category	Men		Women	
	<185% of Poverty Guidelines	≥185% of Poverty Guidelines	<185% of Poverty Guidelines	≥185% of Poverty Guidelines
Screen time	244	194	200	159
Physical activity	17	26	9	17
Travel associated with free time	14	17	12	15
Other free time	112	102	115	112
**Total**	**387**	**339**	**336**	**303**

**Figure 2:**
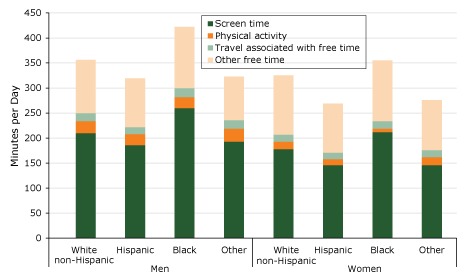
Distribution of free time, in minutes per day, by sex and race/ethnicity (N = 32,048), American Time Use Survey, 2014–2016. Test of equality: *P* = .90 for proportion of screen time by race/ethnicity among men; *P* < .001 for proportion of screen time by race/ethnicity among women; *P* = .07 for proportion of physical activity time by race/ethnicity among men, *P* < .001 for proportion of physical activity time by race/ethnicity among women; *P* < .001 for proportion of screen time or physical activity between men and women. Discrepancies in data between this figure and Table 2 of this article are due to rounding. [A tabular version of this figure is also available.]

Hispanics reported the least amount of free time and screen time (Figure 2). Although the absolute amount of free time and of screen time differed substantially across race/ethnicity among men, the percentage share of screen time was almost identical. Correspondingly, while the absolute time engaged in leisure time physical activity among men was similar across race/ethnicity (Table 2), the ratio of physical activity to free time differed significantly (*P* = .007). Black women reported the most free time and the least physical activity time. Black women spent a mean (SD) of 2.5% (0.3) of their free time on physical activity compared with a mean (SD) of 5.3% (0.2) for white non-Hispanic, 5.0% (0.4) for Hispanic, and 6.7% (0.8) for other women.

**Table:** 

Category	Men				Women			
	Non-Hispanic White	Hispanic	Black	Other	Non-Hispanic White	Hispanic	Black	Other
Screen time	210	186	261	193	178	146	212	146
Physical activity	24	22	22	26	15	12	7	16
Travel associated with free time	16	14	18	17	14	13	15	14
Other free time	106	97	122	87	118	98	121	100
**Total**	**356**	**319**	**423**	**323**	**325**	**269**	**355**	**276**

## Discussion

Americans are less physically active than recommended by guidelines (1). Much less clear is how physical activity fits into people’s lives and how to design effective interventions. Neither surveys of usual activity patterns nor objective measurement with accelerometers have provided a context. Time use data such as those in the ATUS can add this dimension.

Overall, the results for leisure time physical activity confirm patterns seen elsewhere: physical activity levels were lower among women than men; groups with lower income or education compared with those with higher income or educational achievement; older compared with younger respondents; and individuals with obesity compared with those in the normal weight range. The ATUS results also confirm the particularly low levels of leisure time physical activity among black women.

Our definition of free time was intentionally restrictive in that we only considered time to be “free” when activities appear to be entirely discretionary (8). We excluded any self-care, household activities, or family/caretaking from free time even if some could be considered discretionary or leisure time activities (eg, grooming, shopping, playing with children). Time use researchers using less restrictive definitions find larger amounts of free time (11). However, even with a restrictive definition, we found substantial amounts of free time, with no group averaging less than 4.5 hours per day.

In the general press, but also in the public health literature, there appears to be a common belief that Americans have little free time. The American Cancer Society’s guidelines on physical activity state — without providing further evidence — that “reduced leisure time . . . contribute[s] to reduced levels of physical activity” (4). A number of qualitative publications discuss barriers and constraints. One systematic review included 42 studies, just on barriers to physical activity among African American women, and concluded that lack of free time is the most common barrier (12). We found no evidence for those beliefs in nationally representative time use data.

Time use data provide a different perspective to physical activity surveys, accelerometer studies, or qualitative interviews, but have their own set of limitations. For example, time-diary–based estimates of paid work are typically lower than those based on questions about usual hours worked, and respondents tend to give even more inaccurate answers when asked estimate questions about numerous different nonwork daily activities, such as housework (13). Time-diary implicitly constrains responses to sum to 24 hours in a day, and that aids understanding about time trade-offs.

Although the interview prompts for a sequence of activities, activities with short durations are underreported (this is most pronounced for self-care activities). Duration of physical activity is calculated from starting and ending times, but it does not capture the intensity level throughout the period. Therefore, these reports cannot be translated into meeting national guidelines for moderate to vigorous physical activity (14). We only included activities that would be considered exercise, sports, or primarily a leisure time physical activity. Activities where physical activity is not the primary goal but only a secondary benefit, such as playing with children or pet care, would be under caring for others and is not included in the definition of free time. Thus, our analysis is likely to underreport total physical activity.

ATUS is not a good source of information about how much time people spend online because activities are coded on how respondents used the internet. “Ordering groceries online” would be assigned the activity code for “grocery shopping” and it would not be included in free time or screen time. Although there have been attempts to link time use data to energy expenditure (15), we do not attempt to quantify utilitarian physical activity. “Shopping” could mean visiting stores or it could be online ordering, with entirely different implications.

Americans averaged more than 5 hours of free time per day; no subgroup reported having less than 4.5 hours of free time. Substituting at least 20 to 30 minutes with physical activity does seem feasible and would not compromise necessary activities like work, household, family, or self-care (time in those activities is already excluded in our definition of free time). Lack of free time is not responsible for low levels of leisure time physical activity at the population level. Of course, physical activity would have to be convenient and compelling to compete with screen time. Efforts to expand and upgrade settings for physical activity as well as to invest in programming may help to make participation in routine physical activity more attractive.
